# Bleaching and post-bleaching mortality of* Acropora* corals on a heat-susceptible reef in 2016

**DOI:** 10.7717/peerj.8138

**Published:** 2019-12-05

**Authors:** Kazuhiko Sakai, Tanya Singh, Akira Iguchi

**Affiliations:** 1 Sesoko Station, Tropical Biosphere Research Center, University of the Ryukyus, Motobu, Okinawa, Japan; 2 Graduate School of Engineering and Science, University of the Ryukyus, Nishihara, Okinawa, Japan; 3 Geological Survey of Japan, National Institute of Advanced Industrial Science and Technology, Tsukuba, Ibaraki, Japan

**Keywords:** Coral bleaching, *Acropora*, Moderate heat stress, Evolutionary change, Mass transfer, Degree heating week (DHW)

## Abstract

In 2016, global temperatures were the highest on record, and mass coral bleaching occurred world-wide. However, around Sesoko Island, Okinawa, southwestern Japan, the heat stress assessed by degree heating week (DHW) based on local temperature measurements was moderate in 2016; in 1998, DHW was three times higher than in 2016 (10.6 vs. 3.3 in September in respective years). On a reef flat of Sesoko Island where the effect of severe coral bleaching on coral assemblage was monitored in 1998, significant coral bleaching occurred in 2016. Bleaching of the heat stress sensitive *Acropora* corals began in July 2016 on the reef flat as seawater temperature rose. We observed the bleaching and post-bleaching mortality status of individual colonies of *Acropora* spp. in 2016 in fixed plots on the reef flat. In total, 123 *Acropora* colonies were followed for six months after seawater temperature became normal by multiple surveys. At the beginning of September 2016, 99.2% of colonies, were either completely (92.7%) or partially (6.5%) bleached. Of those, the dominant species or species groups were *A. gemmifera* (*Ag*), *A. digitifera* (*Ad*), and tabular *Acropora* (t*A*). For all *Acropora* colonies, the overall whole and partial mortality was 41.5% and 11.4%, respectively. Whole mortality rate differed significantly among species; 72.5%, 17.9%, and 27.8% in *Ag*, *Ad*, and t*A*, respectively. Mortality rates at the end of the surveys were similar in smaller (≤10 cm in diameter) and larger *Ag*, but the former suffered mortality earlier than the latter. Higher survival of smaller colonies was observed only in t*A* (100%), which may be associated with large morphological differences between smaller and larger colonies. Some of the dominant *Acropora* colonies had survived without partial mortality including 15.0% survival of the most vulnerable *Ag* at the end of the surveys. These results suggest that moderate heat stress may have a potential for selecting heat-tolerant genotypes. A longer period of mortality lasting for six months, was observed in *Ag* in addition to immediate whole mortality after bleaching, due to the continuous loss of living tissue by partial mortality. This highlights the need for multiple surveys at least during several months to accurately assess the impact of thermal stress event to corals. In contrast to DHW based on local measurements, DHW obtained from satellite data were similar between 1998 and 2016. Although satellite-based measurement of sea surface temperature is very useful to reveal variations in heat stress at a large spatial scale, temperature should be measured on site when variations at smaller spatial scales are of interest.

## Introduction

Coral reefs develop in warm shallow seas, and support the most biodiverse communities in shallow marine ecosystems (e.g.,  Paulay, 1997). Reef corals, which house symbiotic algae from the family Symbiodiniaceae (LaJeunesse et al., 2018), known as zooxanthellae, in their cells, gain much of their energy from the photosynthates of these zooxanthellae (e.g.,  Muscatine, McCloskey & Marian, 1981). The corals play a core role in coral reef ecosystems by providing habitats (e.g.,  Knowlton, 2001) and food sources for other organisms (e.g.,  Wild et al., 2004). Although the center of the distribution of corals is in warm seas, such as those in the tropics, corals are vulnerable to heat stress. When sea surface temperatures (SSTs) exceed the maximum values in ordinary years by 1 °C in the area where the corals live, the corals may lose their zooxanthellae and bleach (Hoegh-Guldberg, 1999). Bleached corals do not always die, but certain species or genotypes might suffer whole or partial mortality that could allow them to recover by regrowth (Gilmour et al., 2013).

Thermal stress responses are highly heterogenous due to the interaction between extrinsic (environment, stress history and severity) and intrinsic factors (coral halobiont). However thermal stress responses at the same location would probably be governed by intrinsic factors because the extrinsic factors are not likely to vary within the location. Symbiodiniaceae genotype (Berkelmans & Van Oppen, 2006), physiological integration (Swain et al., 2018), heterotrophic capability (Grottoli, Rodrigues & Palardy, 2006), and phylogenetic relationships (Richards, Miller & Wallace, 2013) are some of factors which may govern the thermal stress response across different coral species. Bleaching and mortality variability within the genus *Acropora* have been associated with traits like growth form and colony size (Loya et al., 2001; Baird & Marshall, 2002; Muko et al., 2013). Some of the mechanistic processes which have been proposed explaining such trait specific heat stress tolerance of corals include efficient removal of toxic antioxidants by higher mass flux rates in growth forms with lower volume of space between the branches and smaller colonies (Loya et al., 2001; Van Woesik et al., 2012), protection by hyper pigmentation in smaller colonies (Fabricius, 2006), and differential growth rates (Glynn, 1993; Hoegh Guldberg & Salvat, 1995). Coral mortality following a bleaching event can also depend on the severity and duration of bleaching, and the initial energy stores in the corals (Anthony et al., 2009; Lesser, 2013).

In 1998, mass coral bleaching events occurred world-wide owing to high temperatures associated with the strong El Niño (Hoegh-Guldberg, 1999). This mass coral bleaching also occurred at Okinawa Island, southeastern Japan. At Sesoko Island, which is located in northern Okinawa Island, a severe coral bleaching event occurred with the percentage cover of coral communities (consisting of hard and soft corals) decreasing by 85% and the species density of hard corals by 65% (Loya et al., 2001). Mortality caused by bleaching varied greatly among species; almost all the finely branched corals, such as *Acropora*, *Pocillopora*, and *Seriatopora* spp., suffered whole mortality from bleaching, but many of the massive corals, such as *Porites* spp., survived. Loya et al. (2001) referred to these as the “winners” and “losers” of the coral bleaching event, respectively. However, some of the “loser” species, including *Acropora* spp., recolonized the reef in the 10 years after 1998, and the percentage cover and species density returned to similar levels to before the mass-bleaching event. The new coral community composition had changed, as the relative contribution of *Acropora* spp. such as *A. digitifera*, *A. gemmifera* and tabular *Acropora* increased (Van Woesik et al., 2011). Thus, this reef provided us the opportunity to compare how corals which have recovered after a mass-bleaching event respond to recurrent heat stress. In this paper, we focus on the heat-stress sensitive *Acropora* spp., because *Acropora* corals are potentially the most dominant corals in terms of abundance and percentage cover in many Okinawan reefs, including the present study site (Japanese Ministry of the Environment & Japanese Coral Reef Society, 2004), and contain high species diversity within the genus (Wallace, 1999).

The likelihood of vulnerable species adaptation may be higher under moderate stress rather than extreme thermal stress. Extreme stress such as thermal anomalies can greatly exceed the stress threshold of the vulnerable species, thus causing catastrophic mortality events, resulting in low genetic variability and a higher risk of local extinction (Hoffmann & Hercus, 2000). On the other hand, moderate stress events which are closer to or lower than the stress threshold may drive adaptation by selective survivorship of tolerant genotypes. In 2016, global temperatures were the highest on record, (https://www.climate.gov/news-features/understanding-climate/international-report-confirms-2016-was-third-consecutive-year), and mass-bleaching events occurred world-wide (Hughes et al., 2018a). For example, a mass-bleaching event much more severe than in 1998 occurred on the Great Barrier Reef in 2016 (Hughes et al., 2017). At Sekisei Lagoon in the southern Ryukyu Islands, severe mass coral bleaching also occurred in 2016 (Nakamura, 2017). In contrast to these areas, heat stress during summer at Sesoko Island was weaker in 2016 than in 1998 (see Results), and coral bleaching was observed only at the study site among reefs within 5 km of Sesoko Island (Singh et al., 2019). Thus, the moderate heat stress in 2016 at the study site may have provided the selection pressure for heat stress tolerance. In the present study, our survey focused on the heat sensitive *Acropora* spp. to examine the possibility of selection.

In this paper, we report the effect of moderate heat stress on *Acropora* corals, which were found to be sensitive to heat stress on a reef flat in Sesoko Island where the mortality of corals after a mass-bleaching event was monitored in 1998 (Loya et al., 2001). We also discuss whether moderate heat stress may promote the adaptation of *Acropora* corals. Finally, we highlight the importance of longer monitoring and *in-situ* temperature data measurements in accurately assessing thermal stress responses.

## Materials & Methods

### Study area

This study was conducted on the shallow reef flat in front of Sesoko Station, Tropical Biosphere Research Center, University of the Ryukyus, on the southeastern coast of Sesoko Island, Okinawa, Japan (26°38′N, 127°52′E). The reef flat is approximately 2 m deep at high tide, and the width of the flat from the island offshore is approximately 100 m. Loya et al. (2001) conducted their field survey on the same reef flat.

### Sea surface temperatures (SSTs) and Degree Heating Week (DHW)

Daily SSTs from 1988-2016 were obtained from the Okinawa Prefectural Sea Farming Center, which is 2.5 km away from the study site to the north-northeast. The SST data used in the present study were measured at the same place, at the same time of day as those in Loya et al. (2001). The SST was measured at 8:30 on the surface of a seawater intake well at the farming center. DHW (°C-weeks), which measures the cumulative effect of thermal stress (Liu, Strong & Skirving, 2003) based on MMM_max_ (the mean of the maximum monthly SST from each year in the time period of the climatology; (Donner, 2009; Donner, 2011) over 12 weeks, was calculated for 1998 and 2016 following Kumagai & Yamano (2018):

\begin{eqnarray*}& & DHW= \frac{1}{7} \sum _{i=1}^{84}({\mathrm{HS}}_{maxi},\mathrm{if}:{\mathrm{HS}}_{maxi}\geq \alpha \textdegree C) \end{eqnarray*}

where *i*: day, HS_max__*i*_: HotSpots_max__*i*_ = SST_*i*_ -MMM_max_. The mean of the monthly means of the SST in July and August, when SST is highest in the year, from 1988 to 1997 was employed as MMM_max_, and *α* = 1 °C was used, following previous studies (Liu et al., 2006). In addition to these calculations based on the local SST measurements, DHW at the study site was obtained from NOAA Coral Reef Watch satellite-derived 5 km data (NOAA Coral Reef Watch01, 2018). The DHW data for 1998 and 2016 were downloaded from the NOAA Coral Reef Watch web site (https://coralreefwatch.noaa.gov/satellite/bleaching5km/index.php). The downloaded data were opened by NOAA’s CoastWatch Data Analysis Tool (ver. 3.4.1, https://coastwatch.noaa.gov/cw/user-resources/software-utilities/coastwatch-utilities.html#downloads); the DHW at the study site was obtained on the CoastWatch Data Analysis Tool by pointing at the coordinates of the site. For the period when the value was higher than 0, the DHW was calculated daily from the local data, and for 10-day intervals from the satellite data.

### Survey design and method

**Figure 1:**
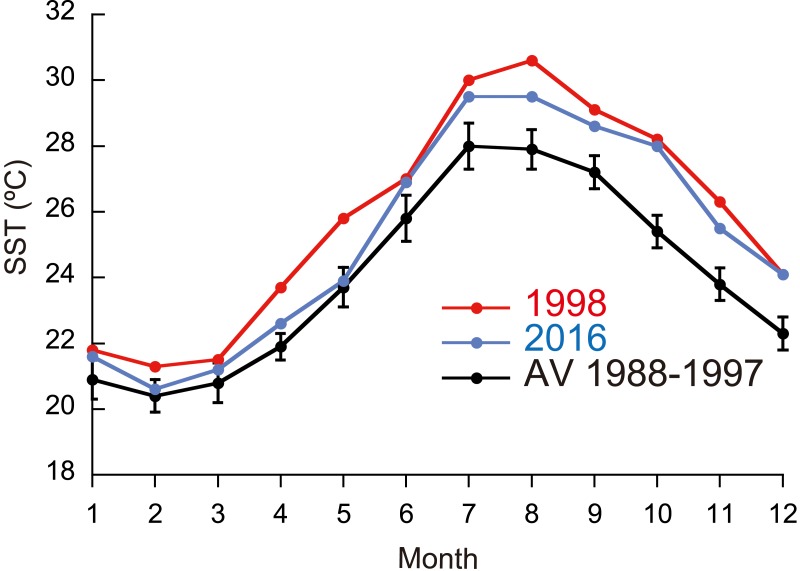
Monthly means of sea surface temperature (SST) in 1998 and 2016, and means for 1988–1997.

The *Acropora* corals at the study site started bleaching from July 2016 (Nishiguchi et al., 2018). Surveys to monitor post-bleaching status began in early September 2016, approximately two months after first bleaching and when SSTs started decreasing (Fig. 1).We monitored all the *Acropora* colonies in four 2 × 2 m fixed plots, which were established approximately 20 m from the edge of the reef to follow the population dynamics of *Acropora* spp. The plots were the same as those at Sesoko Station in our previous study (Singh et al., 2019). During high tide, 1 × 1 m digital images of each 0.5 × 0.5 m area were taken from directly above each plot by a SCUBA diver using a Canon PowerShot S100 in a Canon WP-DC43 underwater housing (Canon Inc., Tokyo, Japan) fitted with a wide-angle lens (INON UWL-H100, × 0.60, INON Inc., Kamakura, Japan). Close-up images of the colonies were also taken when necessary. Images taken for a population dynamics study on April 16, 2016, were used as a record of the pre-bleaching size of the colonies. In these images, a 5 cm scale was placed on each colony for calibration, to facilitate measuring the projected area of the colony on a computer. Post-bleaching surveys were conducted on September 3, 11, and 30, October 10, and November 8 in 2016, and February 4 2017, without using the scales. Except for one small colony (1.9 cm in diameter; Table 1) *Acropora* colonies in the fixed plots were identified to species level from the images in Wallace (1999). Although species with tabular colony morphologies were identified to the species level (*Acropora hyacinthus and A. cytherea*), they were also clumped as tabular *Acropora* because of the difficulty in identifying them by morphology (Suzuki et al., 2016). Hereafter, tabular *Acropora* is referred to as a “species” for simplicity. The projected area (PA) of each colony was measured on the taken images using ImageJ (Schneider, Rasband & Eliceiri, 2012), and the mean diameter (MD) was calculated from the projected area assuming a circle shape for the colony size:

\begin{eqnarray*}& & MD= \left( \sqrt{\mathrm{PA}/\pi } \right) \times 2. \end{eqnarray*}

These measurements were made on images taken on April 2016, i.e., these are initial size.

**Table 1:** Bleaching and mortality status of *Acropora* corals in the fixed plots. The bleaching status was surveyed on September 3, 2016, and mortality status was on February 4, 2017.

Species	Form	*n*	Initial size (diameter, cm)	Bleaching status on Sept. 2016 (%)			Mortality status on Feb. 2017 (%)		
				Complete	Partial	Total	Whole	Partial	Total
*A. aspera*	Arborescent	1	18.0	100.0	0.0	100.0	0.0	0.0	0.0
*A. digitifera*	Corymbose	39	10.0 ± 0.8	97.4	2.6	100.0	17.9	0.0	17.9
*A. gemmifera*	Digitate	40	16.8 ± 1.4	97.5	2.5	100.0	72.5	12.5	85.0
*A. humilis*	Digitate	2	11.7 ± 3.7	100.0	0.0	100.0	50.0	50.0	100.0
*A. nasuta*	Corymbose	4	19.7 ± 5.1	100.0	0.0	100.0	75.0	0.0	75.0
*A. monticulosa*	Digitate	7	25.2 ± 4.9	100.0	0.0	100.0	57.1	42.9	100.0
*A. intermedia*	Arborescent	6	9.5 ± 2.4	83.3	16.7	100.0	33.3	16.7	50.0
*A. robusta*	Arborescent	1	26.3	100.0	0.0	100.0	0.0	100.0	100.0
*A. valida*	Corymbose	3	8.8 ± 1.7	100.0	0.0	100.0	0.0	66.7	66.7
*A. latistella*	Corymbose	1	15.3	100.0	0.0	100.0	0.0	100.0	100.0
Tabular *Acropora*	Tabular	18	20.4 ± 6.3	72.2	27.8	100.0	27.8	0.0	27.8
Unknow		1	1.9	100.0	0.0	0.0	0.0	0.0	0.0
Total		123							

### Interspecies and Intraspecific comparisons

As the bleaching and mortality status did not vary among the plots, all the colonies were pooled in the analyses. Interspecific and intraspecific comparisons were tested only for three species which had high sample size, i.e., *A. digitifera* (*n* = 39), *A. gemmifera* (*n* = 40) and tabular *Acropora* (*n* = 18). Intraspecific comparisons were made by binning the colonies into small (≤10 cm in MD) and large (>10 cm in MD) colonies. The size criterium was set based on an observation by Loya et al. (2001) that some juvenile *Acropora* colonies in the intertidal zone of the present study site remained alive one year after the mass-bleaching event in 1998, and the largest mean diameter of these juvenile colonies was approximately 10 cm. The bleaching (partial or complete) and mortality status (partial or whole) of each colony in all surveys were visually determined from the images. The status of *Acropora* corals were grouped as follows: (1) Partially bleached: entire colony had pale color or basal parts of branches and back side of a colony were colored, (2) Completely bleached: entire colony was white in color, (3) Partial mortality (PM) and bleached: part of a colony was dead and living part was partially or completely bleached, (4) PM and normal: part of a colony was dead and living part had normal color, (5) Normal: colony had normal color without whole or partial mortality. Inter- and intraspecific variations in individual colony trajectories were also examined; and all colonies were divided into following categories: (1) colonies which survived without PM, (2) colonies which suffered PM but survived, (3) colonies which gradually died by PM, and (4) colonies which died without suffering PM. Variation in the timing of mortality was compared between species.

### Statistical analysis

The SST and DHW were statistically compared among years using the Friedman test and Wilcoxon signed-rank test, respectively. Interspecific and intraspecific comparisons were tested only for three species which had high sample size, i.e., *A. digitifera* (*n* = 39), *A. gemmifera* (*n* = 40) and tabular *Acropora* (*n* = 18). Statistical tests were conducted on the bleaching and mortality status measured on September 3 and February 4, respectively. Interspecific bleaching status and mortality rates were compared between species with high sample size using Fisher’s exact test. Intraspecific bleaching status and mortality rates were compared between small (≤10 cm in MD) and large (>10 cm in MD) size classes using Fisher’s exact test. Table 2 shows bleaching and mortality status in two size classes of the three species. Pairwise Fisher’s exact test with “FDR” adjustment method was performed post hoc for all ecological data. All the statistica tests were carried out in JMP® Pro software (ver. 13.2.0, SAS Institute Inc., Cary, NC, 1989-2007) and R v. 3.3.3 (R core team, 2017) using the R packages rcompanion (Mangiafico, 2019), and agricolae ( Mendiburu, 2019).

**Table 2:** Percentage bleaching (September 3, 2016) and mortality (February 4, 2017) rates in two size classes (small, ≤10 cm in mean diameter; large, >10 cm). Number of colonies is shown in parentheses.

Species	Bleaching status				Mortality status				Total number of colonies	
	Partial		Complete		Partial		Whole			
	Small	Large	Small	Large	Small	Large	Small	Large	Small	Large
*A. digitifera*	0.0(0)	6.3(1)	100 (23)	93.8 (15)	0.0 (0)	0,0 (0)	17.4 (4)	18.8 (3)	23	16
Tabular *Acropora*	33.3 (3)	22.2 (2)	66.7 (6)	77.8 (7)	0.0 (0)	0.0 (0)	0.0 (0)	55.6 (5)	9	9
*A. gemmifera*	0.0 (0)	3.6 (1)	75.0 (9)	60.7 (17)	8.3 (1)	14.2 (4)	75.0 (9)	71.4 (20)	12	28

## Results

### SST and DHW

**Figure 2:**
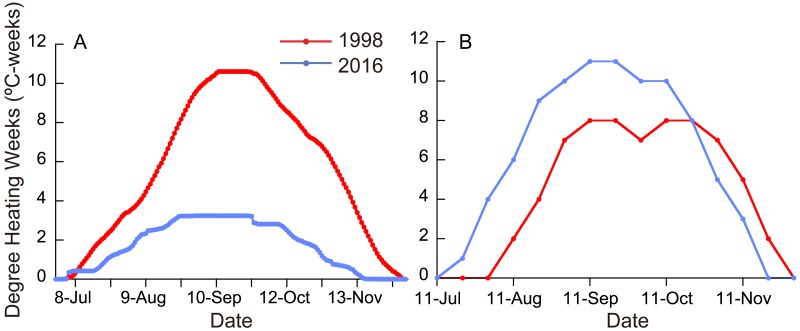
(A) Degree heating weeks (DHW) based on local SST measurements in 1998 and 2016. (B) DHW estimated from satellite data (NOAA Coral Reef Watch01, 2018).

Monthly means of SST for the whole year were significantly different between ordinary years (1988-1997), 1998, and 2016 (Fig. 1; Friedman test; *n* = 12 for each year; *T*_1_ = 22.1; *p* <0.0001), being highest in 1998 and second highest in 2016 (Fisher’s least significant difference test at *α* = 0.05; *t* = 2.07). The daily SST in July and August, the time of year when SST is the highest at the study site, was also highest in 1998 and second highest in 2016 (Friedman test; *n* = 62 for each year; *T*_1_ = 111.9; *p* <0.0001). DHW based on local SST measurements was higher in 2016 than in 1998 for six days in early July, but for the rest of the year was much higher in 1998 than in 2016 (Fig. 2A; Wilcoxon signed-rank test, *n* = 154, *S* =  − 5934.0, *p* <0.0001); the highest DHW in 2016 was 3.3 °C-weeks, while in 1998 it was 10.6 °C-weeks. The DHW was higher than 10 °C-weeks for 27 days from September 5 to October 1 in 1998. DHW estimated from the satellite data (NOAA Coral Reef Watch01, 2018) was quite different from that from the local measurements (Fig. 2B). The satellite-derived DHW was significantly higher in 2016 than in 1998 (Wilcoxon signed-rank test, *n* = 13, *S* = 31.5, *p* = 0.018) and it did not exceed 10 °C-weeks in 1998 (the maximum was 8 in September and October) but did during the same period in 2016.

### Bleaching and mortality status of *Acropora* corals

There were 147 *Acropora* colonies in the plots in April 2016, and none of them had suffered whole mortality by September 3, 2016, when the monitoring of bleached corals was initiated. A typhoon (“Chaba”) hit the study site in October 2016, and 24 *Acropora* colonies disappeared. The vanished colonies were excluded from the analysis, and the remaining 123 colonies were followed until February 2017 (Table 1). Inter- and intraspecies comparisons of bleaching and mortality status were carried out for species with large numbers of colonies; *A. gemmifera* (*n* = 40), *A. digitifera* (*n* = 39), and tabular *Acropora* (*n* = 18).

**Figure 3:**
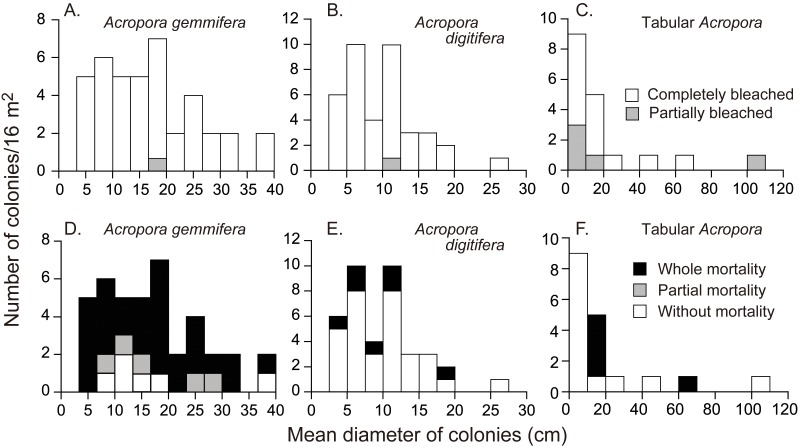
Initial colony size (mean diameter in April 2016), and bleaching and mortality status of *Acropora* species or species groups that appeared in high density (*n* > 17). Bleaching and post-bleaching mortality of *Acropora* corals on a heat-susceptible reef in 2016 (A–C) Bleaching status on September 3, 2016. (D–F) Mortality status on February 4, 2017. Status of *Acropora* colonies were grouped as follows: completely bleached, entire colony was white in color; partially bleached, entire colony had pale color or a colony was almost white but basal parts of branches and back side of a colony were colored; whole mortality, no living parts was in a colony; partial mortality, part of a colony was dead; without mortality, a colony was without whole or partial mortality.

On September 3, all the *Acropora* colonies were partially or completely bleached, except the one small, unidentified colony (Table 1); 6.5% and 92.7% (*n* = 123) of the colonies were partially and completely bleached, respectively. 11.4% (*n* = 114) of completely bleached colonies showed partial mortality (all *A. gemmifera*). The rate of partial bleaching, measured on September 3, 2016, differed significantly among species (Table 1 and; Fisher’s exact probability test; *p* < 0.01). Tabular *Acropora* had a significantly higher proportion of partially bleached colonies (Pairwise Fisher’s exact test, *p* < 0.05), or a smaller proportion of completely bleached colonies, compared to *A. digitifera* and *A. gemmifera*. 2.5% (*n* = 40) of *A. gemmifera* colonies and 2.6% (*n* = 39) of *A. digitifera* colonies were partially bleached, compared to 27.8% (*n* = 18) of tabular *Acropora* colonies. The degree of bleaching was not significantly different between size classes in any of the species (Fig. 3, Fisher’s exact probability test; *p* > 0.2).

**Figure 4:**
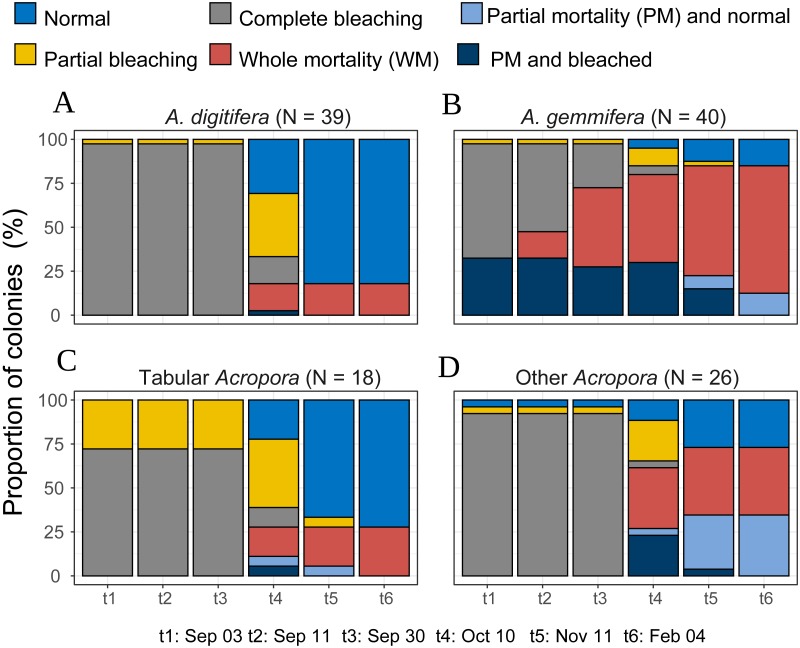
Bleaching and post-bleaching mortality status of *Acropora* colonies (expressed as percentage of colonies) at each survey. (A–C) Species or species group that appeared in high density (*n* > 17) were shown separately. (D) All the species** whose colony number was less than 18 were pooled as “Other *Acropora*”.

*Acropora* colonies had started to recover their colors from October 10, 2016 onwards (Fig. 4). By February 4 when the last survey was conducted, all the surviving *Acropora* corals had returned to a normal color, but 41.5% and 11.4% (*n* = 123) of the colonies suffered whole and partial mortality, respectively. Except one tabular *Acropora,* all the *Acropora* which were partly bleached, had survived, and returned to normal.

The mortality status, measured on February 4, 2017, also differed significantly among the species (Fisher’s exact probability test; *p* < 0.0001) and the species differences were greater than those for bleaching status (Fig. 3; Table 2). Total mortality rates were significantly different among *A. gemmifera*, *A. digitifera*, and tabular *Acropora* (Pairwise Fisher’s exact test, *p* <0.0001). 72.5% and 12.5% ( *n* = 40) of *A. gemmifera* colonies suffered whole and partial mortality, respectively, while only 17.9% (*n* = 39) of *A. digitifera* and 27.8% (*n* = 18) of tabular *Acropora* colonies suffered whole mortality. Partial mortality was not observed in these two species. The intraspecific differences in mortality status between size classes was significant only in tabular *Acropora* (Table 2; Fig. 3; Fisher’s exact probability test, *p* = 0.03), where all the colonies which suffered whole mortality were larger than 10 cm in mean diameter.

### Inter-species variations of individual *Acropora* trajectories

Temporal patterns of *A. gemmifera* differed considerably compared to *A. digitifera* and tabular *Acropora* (Fig. 4). In the first survey, 32.5% (*n* = 40) of *A. gemmifera* showed partial mortality. Whole mortality of *A. gemmifera* also started approximately a month earlier than the other two species (Table 3). Furthermore, whole mortality rates of *A. gemmifera* continued to rise until February 2017.

**Table 3:** Approximate time of whole mortality of *Acropora* colonies. Size classes are the same with in Table 2. Because significant difference in the whole mortality time was observed only for *A. gemmifera*, all other *Acropora* were pooled. Time until whole mortality was expressed as months after the first *Acropora* bleaching report at the study site in 2016 (Nishiguchi et al., 2018). Number of colonies is shown in parentheses in whole mortality rate.

Date of observation	Approximate time until whole mortality (months)	Whole mortality rate (%)		
		Small *A. gemmifera* (*N* = 12)	Large *A. gemmifera* (*N* = 28)	Other *Acropora* (*N* = 83)
Sept. 03, 2016	2.0	0.0	0.0	0.0
Sept. 11, 2016	2.5 to 3.0	33.3 (4)	7.1 (2)	0.0
Sept. 30, 2016	3.0 to 3.5	66.7 (8)	35.7 (10)	0.0
Oct. 10. 2016	3.5 to 4.0	75.0 (9)	39.3 (11)	21.7 (18)
Nov. 11, 2016	4.0 to 5.0	75.0 (9)	57.1 (16)	25.3 (21)
Feb. 4, 2017	5.0 to 8.0	75.0 (9)	71.4 (20)	26.5 (22)

On further examination of individual colony trajectories we found that a large proportion of both small (41.7%; *n* = 12) and large (57.1%; *n* = 28) *A. gemmifera* colonies suffered continuous partial mortality and eventually suffered whole mortality (Table S1). This occurred only for one *A. digitifera* (PA = 118 cm^2^) and two tabular *Acropora* colonies (PA = 123 and 213 cm^2^). All three of these colonies eventually suffered whole mortality by February 2017 (Table 3).

### Intra-species variations of individual *A. gemmifera* trajectories

The majority of both small and large *A. digitifera* and tabular *Acropora* colonies survived without partial mortality (Table 2). Survival patterns of small and large *A. gemmifera* were similar; 16.7% of small (*n* = 12) and 14.4% of large (*n* = 28) colonies were alive without suffering any partial mortality, and 8.3% of small and 14.2% of large *A. gemmifera* colonies remained alive after suffering partial mortality. The only difference within *A. gemmifera* was that 33.3% of small and 14.3% of large colonies suffered whole mortality without partial mortality.

**Figure 5:**
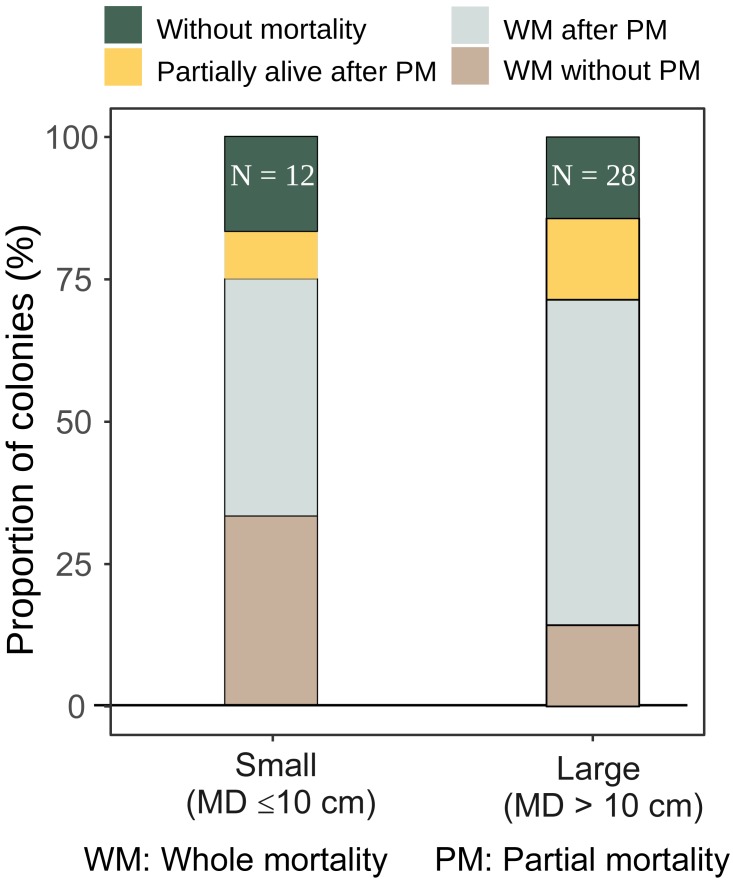
Fate of small and large *A. gemmifera* colonies (expressed as percentage of colonies) at the last survey (February 2017).

Although by the last survey, both small and large colonies of *A. gemmifera* had similar whole mortality rate (75.0% and 71.4%, Fig. 5), the timing of mortality was different between size classes. Smaller *A. gemmifera* colonies stopped whole mortality 3.5 months after first bleaching, while larger *A. gemmifera* colonies continued to suffer whole mortality till the last survey, or six months after first bleaching (Table 3). Large *A. gemmifera* colonies which suffered whole mortality later, were those initially experiencing partial mortality (Table S1).

## Discussion

Moderate heat stress may facilitate evolutionary changes towards higher heat tolerance in heat sensitive coral species. The highest DHW at the study site, based on the local measurement of SST during summer (July and August) in 1998, was 10.6 while that in 2016 was 3.3. The majority of the large *Acropora* species at the study site bleached and died off from the severe heat stress in the summer of 1998 (Loya et al., 2001). Hence, the selection of heat stress-tolerant genotypes in these colonies was not possible in 1998 at the study site. In contrast, although all *Acropora* bleached, whole mortality was 17.9% and 27.8% in *A. digitifera* and tabular *Acropora* in 2016, respectively. Whole mortality was much higher in *A. gemmifera*, but 15.0% of them survived without suffering partial mortality. Survival of even a few *Acropora* colonies may indicate selection for heat stress-tolerant genotypes including ability to conduct epigenetic changes against heat stress (e.g., Putnam et al., 2017) by the moderate heat stress observed in 2016. In the Great Barrier Reef, although the cumulative heat stress in 2017 was similar to or even higher than in 2016, coral bleaching in 2017 was less severe than that in 2016 (Hughes et al., 2019). A plausible hypothesized mechanism for the lower bleaching in 2017 was that the proportion of more heat-tolerant colonies had increased in the northern Great Barrier Reef (Hughes et al., 2019). In the Great Barrier Reef, mortality of heat-stress sensitive coral taxa such as *Acropora* was high, but on average was not as high as 100% (Extended Data Fig. 4 in Hughes et al., 2018b). After the 1998 mass-bleaching event, almost all the *Acropora* colonies at the study site died except for small colonies in the intertidal zone (Loya et al., 2001). Hence, the *Acropora* colonies that appeared in the permanent plots most likely originated from larvae that settled at the study area after the 1998 mass-bleaching event. Under high seawater movement or a turbid environment, corals may survive after heat stress (e.g., Nakamura & Van Woesik, 2001; Guest et al., 2016; Singh et al., 2019). For example, many *Acropora* colonies survived after 1998 below 8 m in a high seawater movement environment at Bise, which is located 8 km north of the study site (Nakamura & Van Woesik, 2001). Such reefs, with a mix of heat-tolerant and vulnerable genotypes, were probably the source reefs that contributed larvae to the study site, resulting in genotypic diversity in terms of heat tolerance in the *Acropora* corals at the study site by 2016.

There are at least three hypotheses to explain interspecific variation in the bleaching and post-bleaching mortality rates within the genus *Acropora* corals. All the colonies of the most abundant three species, i.e., *A. gemmifera*, *A. digitifera*, and tabular *Acropora*, were either completely or partially bleached in the summer of 2016. The rate of whole and partial mortality after bleaching was more strikingly different among species than the bleaching rate.

**Figure 6:**
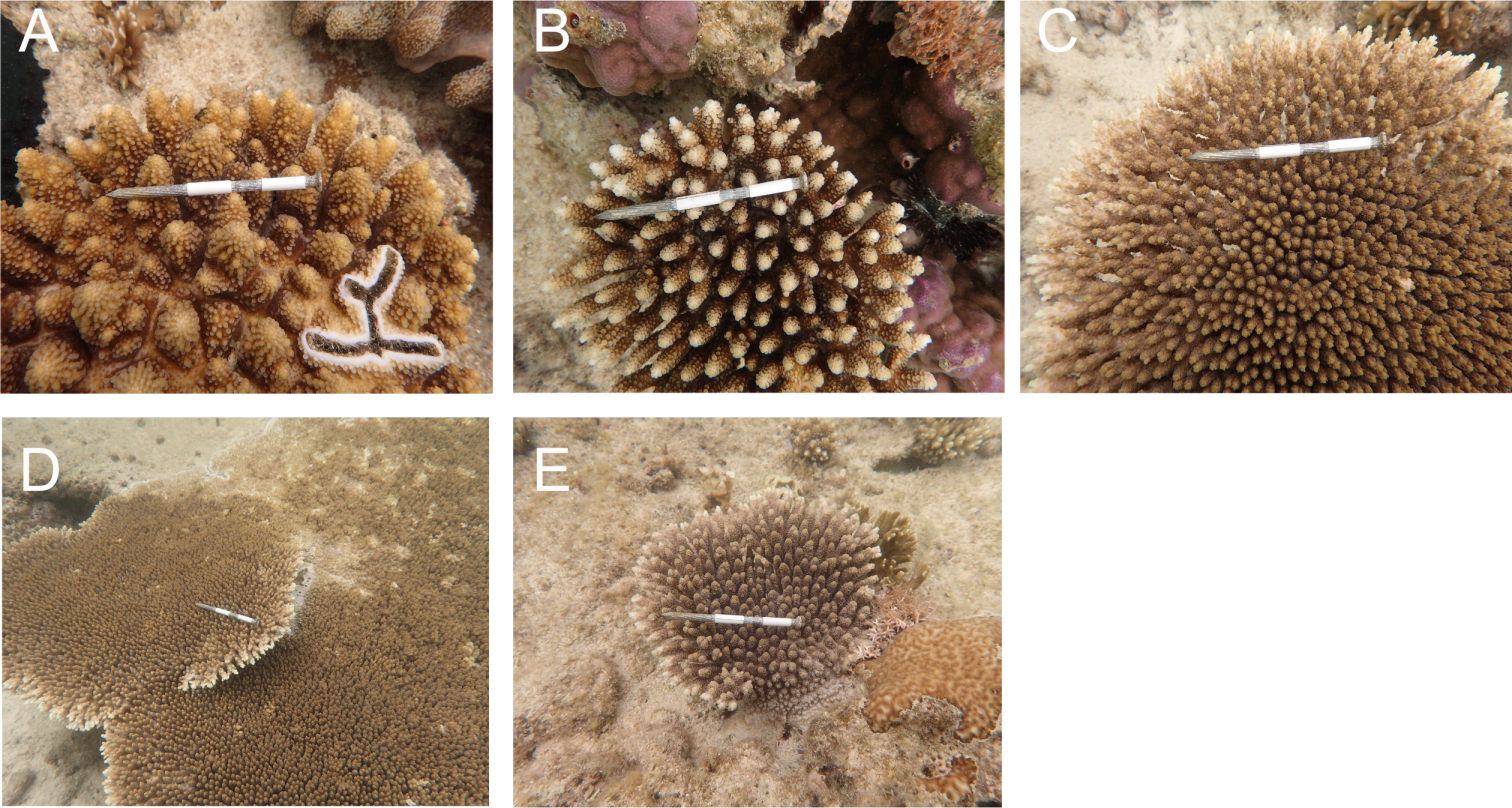
(A) *A. gemmifera*. (B) *A. digitifera*. (C–E) Tabular *Acropora*. Distance between both end of plastic tape s is 5 cm.

The first hypothesis is the mass transfer hypothesis (Patterson, 1992). The mortality rate was the highest in the *A. gemmifera*. The colony morphologies of *A. gemmifera* and *A. digitifera* at the study site were digitate and corymbose (Table 1). Mass flux theory can potentially explain our results which predicts that corals with a high interstitial domain to boundary domain ratio (*I* d:*B* d) have lower rates of passive diffusion than those with a lower ratio, and the former would be more vulnerable to heat stress (Van Woesik et al., 2012). The *I* d:*B* d ratio of *A. gemmifera* looked to be the highest of the three species (Figs. 6A and 6C). Intraspecific variations of mortality owing to colony size may also be attributable to the mass transfer hypothesis. In tabular *Acropora*, higher survival rates were observed in smaller sized colonies (S, ≤ 10 cm in diameter) compared to larger (L, >10 cm in diameter). This observed difference in mortality rate may be explained by the mass transfer hypothesis, because due to the flatter shape of S colonies and overlapping “tables” in L colonies which possessed higher *I* d:*B* d ratios (Figs. 6D and 6E).

The second hypothesis is that initial energy stores can influence the risk of mortality after bleaching (Anthony et al., 2009). *Acropora* corals started to bleach approximately a month after the mass spawning event. The amount of energy invested in reproduction may vary among species. Therefore, it is possible that the three species had different initial energy stores when they started to bleach, resulting in species specific mortality. The third hypothesis is that differences in bleaching and mortality rates can be explained by differences in genus or species of the symbiotic algae (family Symbiodiniaceae; LaJeunesse et al., 2018). For example, it was reported that corymbose colonies of *Acropora millepora* with genetically distinct symbiotic algae showed different bleaching responses to heat stress (Berkelmans & Van Oppen, 2006). Thus, genotyping of the Symbiodiniaceae in the *Acropora* species at the study site should be explored in future studies.

In addition, other hypotheses have been put forward to explain differential interspecific susceptibility to heat stress. One of them stated that, by taking phylogenetic relationships into account, species with higher coloniality or physiological integration are more resistant to bleaching (Swain et al., 2018). However, this may not be the case in *Acropora* spp., since all *Acropora* spp. appeared to have similar levels of coloniality. Instead phylogenetic relationships may explain the mortality response among the genus *Acropora* (Richards, Miller & Wallace, 2013). Another involves interspecific differences in tissue thickness (Hoegh-Guldberg, 1999), but this is not applicable here; the most susceptible species *A. gemmifera* and the less susceptible *A. digitifera* had very similar tissue thickness at the study site (Loya et al., 2001). Some authors argued that coral species with high growth rates might be less resistant to heat stress (Glynn, 1993; Hoegh Guldberg & Salvat, 1995); however, growth was not found to be related to susceptibility at the study site (Singh et al., 2019). Basically, several intrinsic factors can drive thermal stress response but studies comparing the above factors in multiples species within the genus *Acropora* are scarce. The genus *Acropora* is among the most dominant and diverse coral groups in Indo-pacific reefs and their response to moderate thermal stress is bound to be variable which can affect future coral reef communities. It is thus critical to identify and partition the roles of different factors in driving the thermal stress response within the genus *Acropora*.

Earlier increases in the whole mortality rates of S colonies compared to L colonies in *A. gemmifera* may be attributable to the relatively small energy storage in the smaller colonies. The whole mortalities of S of *A. gemmifera* were greater than L colonies until September 30 (t3) and stopped increasing after October 10 (t4), or 37 days after the SST started decreasing. In contrast, the partial and whole mortalities of L colonies continued to increase until the end of the study period, or 154 days after the decrease in SST, resulting in similar whole and partial mortalities in S and L colonies at the end of the survey. L colonies are considered to have larger energy stores than smaller colonies. For example, experimentally generated lesions on the massive coral *Favia* (currently *Dipsastraea*; Budd et al., 2012) *favus* healed faster in larger than in smaller colonies (Oren et al., 2001). The healing of the lesions by the massive corals was presumed to be due to the translocation and utilization of photosynthetic energy sources within the colonies (Oren et al., 2001). The faster healing in the larger massive corals suggests that, in general, the energy storage is relatively higher in larger than in smaller colonies. In the present study, L colonies of *A. gemmifera* suffered less whole mortality than S colonies until t3, which may be due to their relatively larger energy storage capability. However, after t4, whole mortality increased in L colonies, and the mortality rate was similar in the L and S colonies at the end of the survey, suggesting that only heat-tolerant genotypes survived, irrespective of colony size.

The present study highlights the need for multiple surveys over at least six months to assess the fate of corals after the bleaching. We observed two patterns in whole mortality in multiple surveys during the period of six months after bleaching. One was gradual loss of tissue, or increase in partial mortality, while the other was whole mortality without suffering partial mortality. The former was observed in *A. gemmifera* while latter in *A. digitifera* and tabular *Acropora.* Most of the *Acropora* which suffered partial mortality also suffered whole mortality later. This response was independent from size. Very few *Acropora* spp. (consisting of all morphologies) could survive after suffering partial mortality. If we had surveyed the bleaching response just three months after seawater temperature became normal, we would have erroneously concluded that all species had similar mortality levels.

The direct measurement of SST is indispensable to assess heat stress for corals at a small spatial scale. Satellite-based estimation of SST is a very powerful tool to assess heat stress for corals at large spatial scales. NOAA conducts daily Coral Bleaching Heat Stress Monitoring at a global scale using satellite data (https://coralreefwatch.noaa.gov/satellite/index.php). For example in the Caribbean, comparisons of the NOAA Coral Reef Watch’s DHW with bleaching severity data from field surveys demonstrated a predictive relationship between the satellite-derived DHW and bleaching intensity, at a spatial scale of 4,000 km (Eakin et al., 2010). As another successful example, satellite-based DHW showed a strong positive relationship with the percentage of bleached corals recorded underwater in the central and northern Great Barrier Reef at a 1,200-km spatial scale (Hughes et al., 2017). In contrast to such studies on large spatial scales, the present study compared bleaching intensity and DHW on the same reef between 1998 and 2016. Probably owing to an error associated with the satellite measurements at very small spatial scales, the discrepancy in DHW between the local direct-measurements and the satellite measurements was very large in this study. If only the satellite-based DHW was used in the present bleaching data set, we might have erroneously concluded that *Acropora* corals at the study site became more heat-tolerant in 2016 than they were in 1998.

## Conclusions

In this study, we monitored the bleaching and post-bleaching mortality status of heat-stress sensitive *Acropora* spp. at Sesoko Island in 2016. Our results suggest the potential for selection by moderate heat stress for heat-tolerant genotypes within these species, which may lead to evolutionary changes in coral populations. This study indicates the usefulness of measuring temperature and examining the colony sizes and morphologies of coral populations at small spatial scales. The present study also indicates the need for multiple surveys over longer time period to assess the fate of corals after the bleaching.

##  Supplemental Information

10.7717/peerj.8138/supp-1 Table S1 Accumulated number of dead colonies of *Acropora gemmifera* occurred by gradual loss of live tissue after partial mortality (PM)

10.7717/peerj.8138/supp-2 File S1 Raw data of bleaching (September 3, 2016) and mortality (February 4, 2017) status of *Acropora* corals in the fixed plots**

10.7717/peerj.8138/supp-3 File S2 Changes in projected area of colonies of *Acropora gemmifera* from September 2016 to February 2017

10.7717/peerj.8138/supp-4 File S3 Daily value of Degree heating week in 1998 and 2016 from local measurements of sea surface temperature by Okinawa Prefectural Sea Farming Center

## Additional Information and Declarations

### Data Availability

The following information was supplied regarding data availability: The raw data are available as Supplemental Files.
