# Supplementary material for: Bleaching and post-bleaching mortality of Acropora corals on a heat-susceptible reef in 2016
Source: PeerJ. 2019 Dec 5;7:e8138. doi: 10.7717/peerj.8138 (PMC6899343; doi:10.7717/peerj.8138)
Supplement: Table S1 [file peerj-07-8138-s001.docx]

| Date of mortality observation | Died without PM | Died after PM | Died without PM | Died after PM |
| --- | --- | --- | --- | --- |
|  | Small (N =12) | | Large (N = 28) | |
| Sept. 03, 2016 | 0 | 0 | 0 | 0 |
| Sept. 11, 2016 | 2 | 2 | 0 | 2 |
| Sept. 30, 2016 | 3 | 5 | 4 | 6 |
| Oct. 10. 2016 | 4 | 5 | 4 | 7 |
| Nov. 11, 2016 | 4 | 5 | 4 | 12 |
| Feb. 4, 2017 | 4 | 5 | 4 | 16 |
